# Nocardia otitidiscaviarum Pneumonia and Empyema in a Woman With Colon Adenocarcinoma

**DOI:** 10.7759/cureus.72663

**Published:** 2024-10-29

**Authors:** Javier Cabrera-Sanchez, Hermes Tejada, Eloy E Ordaya

**Affiliations:** 1 Medicine, Universidad Peruana Cayetano Heredia, Lima, PER; 2 Medicine, Clínica Médica Cayetano Heredia, Lima, PER; 3 Oncology, Clínica Médica Cayetano Heredia, Lima, PER; 4 Infectious Diseases, Henry Ford Health, Michigan, USA

**Keywords:** antimicrobial susceptibility pattern, colon adenocarcinoma, empyema, necrotizing pneumonia, nocardia otitidiscaviarum

## Abstract

We report a case of necrotizing pneumonia and empyema in a woman with colon adenocarcinoma, which developed shortly after receiving chemotherapy. A chest tube was placed, and analysis of the pleural fluid identified *Nocardia otitidiscaviarum*. The patient underwent pleural decortication via video-assisted thoracoscopy and was treated with a combination of trimethoprim-sulfamethoxazole and imipenem/cilastatin. After four weeks of treatment, the patient was discharged with oral trimethoprim-sulfamethoxazole, with no recurrence noted during follow-up. *Nocardia otitidiscaviarum* is an uncommon pathogen that can cause both localized and disseminated disease, similar to other *Nocardia* species. Its susceptibility to various antibiotics is variable, making susceptibility testing essential when available, along with close monitoring for potential medication side effects.

## Introduction

*Nocardia* is a genus of filamentous, gram-positive, aerobic bacteria commonly found in soil, vegetation, and organic matter [1]. The epidemiology of *Nocardia spp*. varies throughout the world, with the most common species in the United States being *Nocardia nova*, *Nocardia cyriacigeorgica*, and *Nocardia farcinica* [2]. *Nocardia* species predominantly affect immunocompromised individuals, particularly those receiving steroids and chemotherapy, commonly involving the lungs, skin, and, less frequently, the brain [3-5]. Cultures of respiratory, blood, or tissue samples are used to diagnose *Nocardia spp.* infections, but speciation is frequently attained via 16S RNA analysis or matrix-assisted laser desorption ionization-time of flight mass spectrometry (MALDI-TOF) [1]. Even though most clinically relevant *Nocardia spp.* are susceptible to trimethoprim-sulfamethoxazole, imipenem, and amikacin, higher rates of resistance to first-line antibiotics have been reported in less common species [1,6]. *Nocardia otitidiscaviarum* is a rare species that has not been previously reported in Latin America. Here, we report a case of necrotizing pneumonia and empyema caused by *Nocardia otitidiscaviarum.*

## Case presentation

A 51-year-old female from Peru presented to the emergency department with thoracic and shoulder pain. Three days before the presentation, she started to complain of right shoulder pain that progressively increased in intensity over the following days. On the day of presentation, her shoulder pain became more intense, and she reported right-sided pleuritic chest pain, prompting her to seek medical care. She denied fever, cough, shortness of breath, or headache. Her medical history was significant for rheumatoid arthritis previously treated with steroids (not currently on treatment), asthma, and colon adenocarcinoma with liver and brain metastases, managed with hemicolectomy, brain radiotherapy, and chemotherapy with FOLFOX-6 and bevacizumab. Sixteen milligrams of dexamethasone were given before each chemotherapy session. She had received her last chemotherapy cycle two days before symptom onset.

On presentation, her temperature was 37.2°C, blood pressure was 140/90 mmHg, heart rate was 99 beats/min, respiratory rate was 26 breaths/min, and oxygen saturation was 96% on room air. On exam, she appeared uncomfortable, with absent breath sounds in the right lung base. There was no swelling, erythema, or warmth in the right shoulder. The rest of the exam was unremarkable.

**Figure 1:**
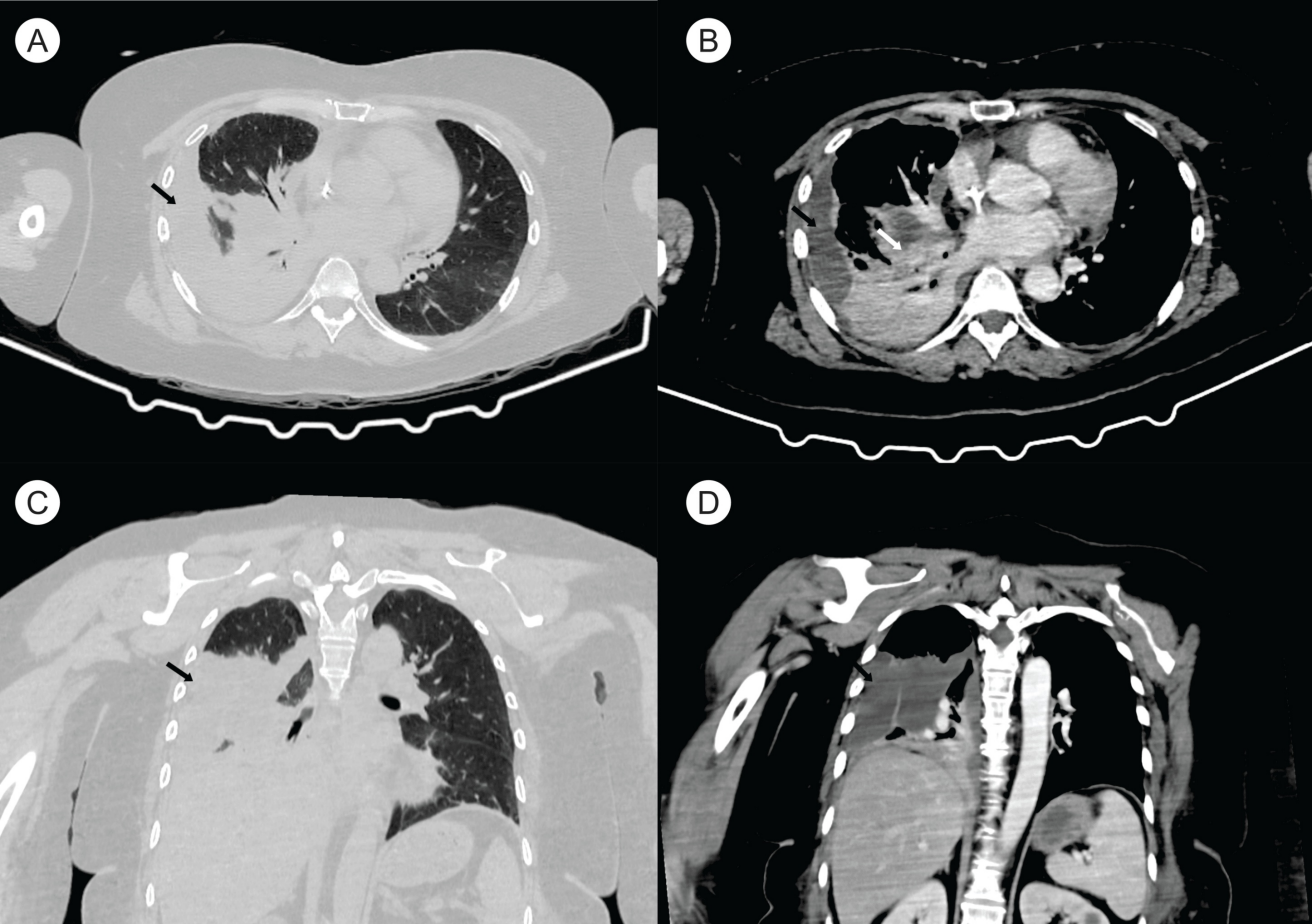
Chest CT done on admission showing a right pleural effusion Pleural effusion (black arrows in A-D) and a consolidative lesion (white arrow in B) are seen in the right lung. (A) Axial chest CT with lung window. (B) Axial chest CT with mediastinal window. (C) Coronal view with lung window. (D) Coronal view with mediastinal window.

Laboratory tests showed leukocytosis at 14,000/mm³ (normal value: 4,000-11,000/mm³), C-reactive protein at 200 mg/L (normal value <5 mg/dL), and procalcitonin at 20 ng/mL (normal value <0.5 ng/mL). A D-dimer test was negative. The patient tested negative for HIV. A shoulder X-ray was unremarkable, and an ultrasound showed mild synovitis. A right shoulder CT scan showed no bone lesions, but lung imaging revealed a right pleural effusion (Figure 1), which led to her admission.

A few hours after being transferred to the medical ward, she developed fever, dyspnea, and hypoxia (oxygen saturation 85%), requiring supplemental oxygen and chest tube placement. The pleural fluid was sent for analysis (Table 1), and the patient empirically received meropenem and vancomycin.

**Table 1:** Fluid analysis from the first pleural fluid sample *Bacterial culture from the first sample was negative. However, subsequent samples were positive.

Pleural fluid study	Results	Reference range
Leucocytes	820/uL(79% neutrophils)	<500/uL
Red blood cells	9,973/uL	<100/uL
Proteins	3.45 g/dL	1-3 g/dL
Glucose	37 mg/dL	50-90 mg/dL
Dehydrogenase lactate	490 IU/L	Not available
Adenosine deaminase	49 IU/L	<10 IU/L
Bacterial culture	Negative*	Negative
Mycobacterial culture	Negative	Negative
Fungal culture	Negative	Negative
Mycobacterium tuberculosis Gene-Xpert	Negative	Negative
Cytology	Negative for malignant cells	Negative
Block cell	Negative	Negative

**Figure 2:**
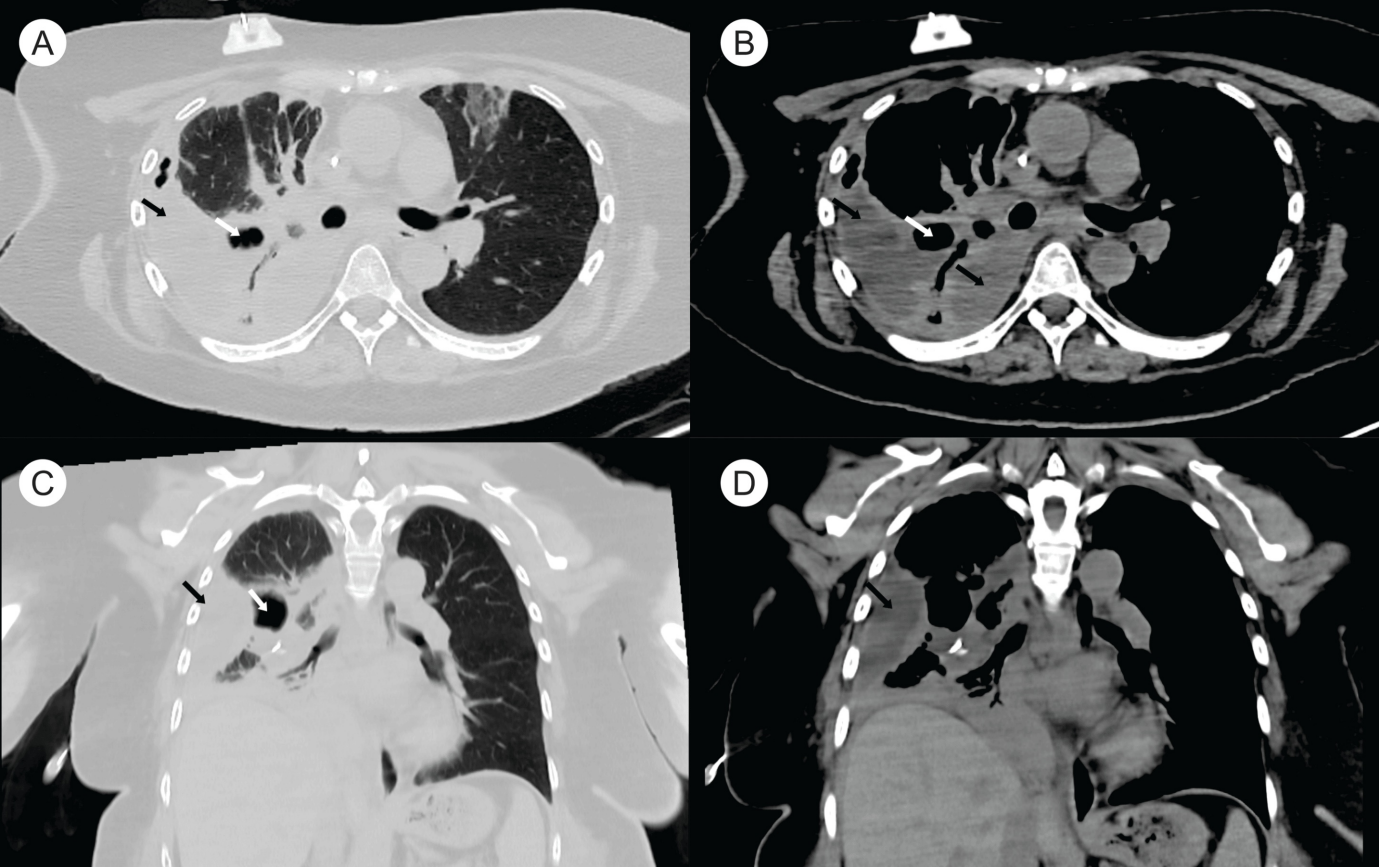
Chest CT after one week of treatment Chest CT after one week of chest drainage with a thoracostomy tube and empiric antibiotics showing incomplete resolution of the empyema (black arrows) and necrotic lung zones (white arrows). (A) Axial chest CT with lung window. (B) Axial chest CT with mediastinal window. (C) Coronal view with lung window. (D) Coronal view with mediastinal window.

The initial pleural fluid was hemorrhagic, but subsequent drainage yielded purulent fluid that was sent for cultures. The patient remained febrile despite seven days of broad-spectrum antibiotics and a patent right-sided chest tube. Blood cultures and serum galactomannan tests were negative. A repeat chest CT scan showed persistent loculated effusions and hypodense lesions (Figure 2).

Surgical management was deemed necessary, and the patient underwent pleural decortication via video-assisted thoracoscopy and partial lobectomy. Surgical findings included loculated empyema complicated by lung entrapment, pulmonary abscesses, and necrosis.

Three days post-surgery, the microbiology lab reported the growth of a filamentous, acid-fast organism in the pleural fluid (second sample), raising suspicion of *Nocardia spp*. MALDI-TOF confirmed the presence of *Nocardia otitidiscaviarum*, later isolated from intraoperative cultures. Susceptibility testing was not performed due to laboratory limitations. Fungal and mycobacterial cultures were negative. Lung and pleural pathology showed extensive necrosis and inflammatory cell infiltrates without granuloma formation or malignant cells. A head CT with contrast showed sequelae of the previously documented brain metastasis but no new lesions suggestive of cerebral nocardiosis.

**Figure 3:**
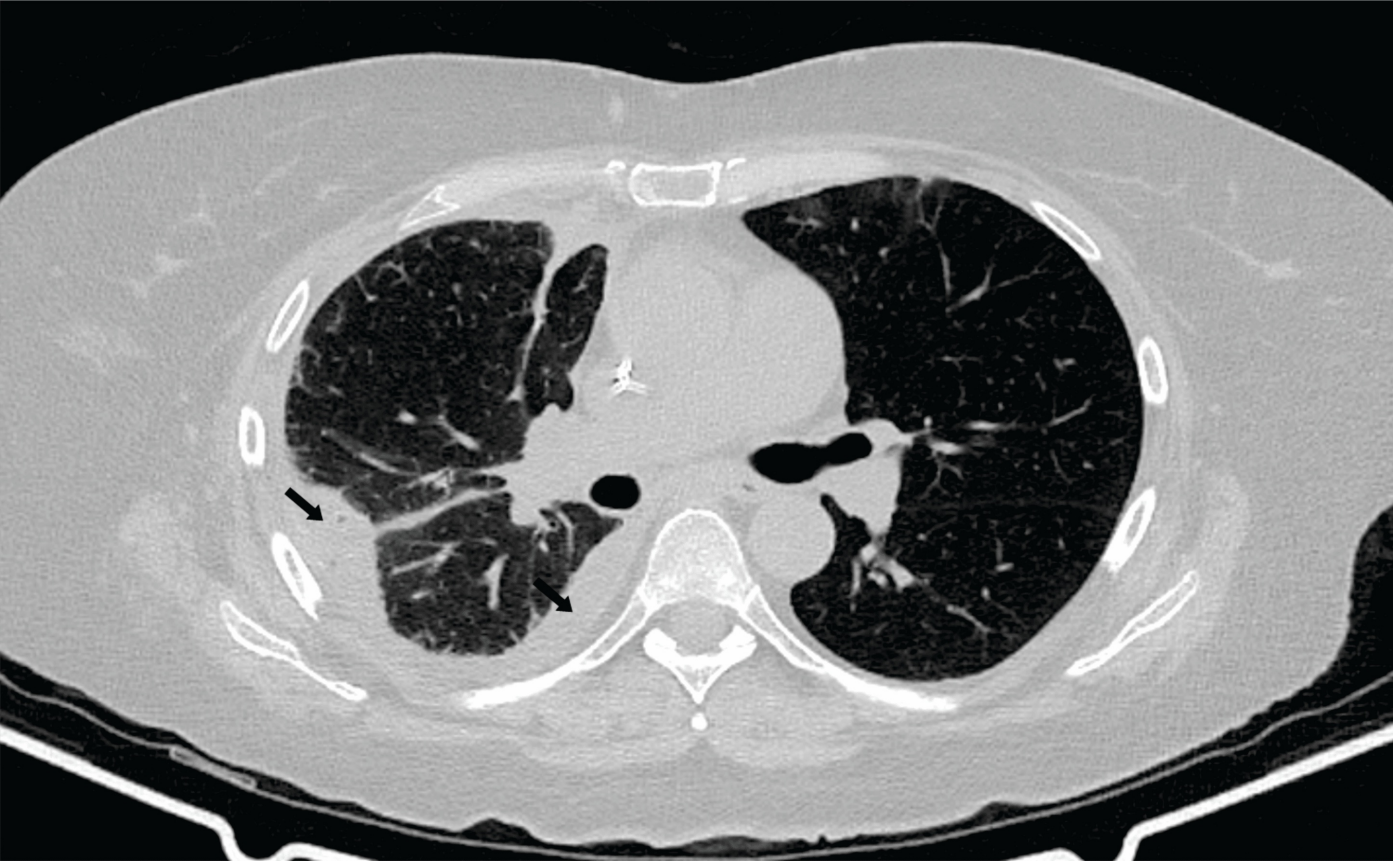
Chest CT one month after discharge Chest CT was performed one month after discharge. A decrease in effusion size (black arrows) is seen with no new consolidations.

Given the availability of antimicrobials and potential toxicities associated with alternative options in this patient, who had a history of chemotherapy-induced pancytopenia and risk factors for acute kidney injury, her treatment was switched to intravenous trimethoprim-sulfamethoxazole and imipenem/cilastatin, with consideration of adding linezolid or amikacin if clinical deterioration occurred. The patient received four weeks of dual intravenous antibiotic therapy, followed by oral trimethoprim-sulfamethoxazole for six months. A follow-up chest CT after discharge showed a decrease in pleural effusion without new consolidations (Figure 3). No new symptoms or recurrence were reported during follow-up.

## Discussion

We present the case of an immunocompromised woman diagnosed with nocardiosis following recent chemotherapy. To our knowledge, this is the first documented case of *Nocardia otitidiscaviarum* infection in Latin America.

The distribution of *Nocardia* species causing human disease varies geographically (Table 2) [6-18]. In the United States and Canada, the most common species are *Nocardia nova, Nocardia cyriacigeorgica*, and *Nocardia farcinica*. In contrast, *Nocardia otitidiscaviarum* accounted for only 0.9% to 6% of nocardiosis cases reported in these countries [7,8]. This species was also rarely reported in a study conducted in Spain, constituting only 3.1% of 1,119 *Nocardia* isolates [9]. In another study conducted in China and Japan, *Nocardia otitidiscaviarum* represented 5.9% and 7% of cases, respectively. Nevertheless, *Nocardia otitidiscaviarum* was among the most prevalent species in two studies conducted in India and Iran [15,16]. Data on *Nocardia* species distribution from Latin America is scarce, but most studies report *Nocardia farcinica, Nocardia brasiliensis*, and *Nocardia asteroides* as the most frequent Nocardia species in this region [17,18].

**Table 2:** Microbiological studies showing the percentage of Nocardia infections caused by Nocardia otitidiscaviarum

First author	Year	Country	Number of *Nocardia* isolates	The most common isolated *Nocardia* species	Percentage of *N. Otitidiscaviarum* (%)	
						Hamdi [2]	2020	USA	2091	*N. nova* (21.6%), *N. cyriacigeorgica* (16,8%),* N. farcinica *(15.3%), *N. brasiliensis* (10.7%)	1.4	
Harris [7]	2021	USA	110	*N. brasiliensis *(18%), *N. farcinica* (18%), *N. nova* (15%), *N. cyriacigeorgica *(14%)	0.9	
Mctaggart [8]	2015	Canada	149	*N. farcinica* (24%), *N. nova* (19%) *N. cyriacigeorgica* (13%), *N. abscessus* ( 6%)	4	
Valdezate [9]	2017	Spain	1119	*N. cyriacigeorgica *(25.3%), *N. nova* (15%), *N. abscessus* (12.7%), *N. farcinica *(11.4%)	3.1	
Wei [10]	2017	China	28	*N. cyriacigeorgica* (25.3%), *N. nova* (15%), *N. abscessus* (12.7%),* N. farcinica *(11.4%)	3.5	
Wang [11]	2022	China	441	*N. farcinica *(9.9%), *N. cyriacigeorgica* (28.6%), *N. abscessus* (6.6%), *N. otitidiscaviarum* (5.9%)	5.9	
Lu [12]	2020	China	27	*N. otitidiscaviarum* (40.7%), *N. cyriacigeorgica* (25.9%), *N. brasiliensis* (11.1%), *N. asteroides* (7.4%)	40.7	
Toyocawa [13]	2021	Japan	153	*N. farcinica* (25%),* N. cyriacigeorgica* (18%), *N. brasiliensis* (9%), *N. nova* (8%)	7	
Gnanam [14]	2020	India	22	*N. farcinica* (45%), *N. cyriacigeorgica* (18%), *N. otitidiscaviarum* (14%),* N. amikacinitolerans* (9%)	14	
Kudru [15]	2021	India	48	*N. otitidiscaviarum* (40.7%), *N. cyriacigeorgica* (25.9%), *N. brasiliensis* (11.1%),* N. asteroides* (7.4%)	40.7	
Rahdar [16]	2021	Iran	23	*N. cyriacigeorgica* (30.4%), *N. otitidiscaviarum* (17.4%),* N. asteroides *(13%), *N. Ignorata* (8.7%)	17.4	
Sánchez-Herrera [17]	2012	Mexico	18	*N. farcinica *(61%), *N. brasiliensis *(39%)	0	
Baio [18]	2013	Brasil	48	*N. asteroides* (65.4%), *N. brasiliensis* (11.5%), *N. farcinica *(7.7%), *N. pseudobrasiliensis* (3.8%)	0	

The patient's chemotherapy may have contributed to an increased susceptibility to Nocardia infection, as reported in previous cases, although the degree of immunosuppression with FOLFOX may not be as severe as that caused by other therapies [4-5]. The patient’s active malignancy may also exert immunomodulatory effects of variable intensity, potentially heightening the risk of Nocardia infection. It is important to note that she was HIV-negative and did not have a history of recurrent infections. Additionally, it is worth noting that up to one-third of nocardiosis cases occur in patients without any clearly identifiable immunosuppressive condition, suggesting that overt immunosuppression is not always necessary for the development of this infection [1].

In our literature review, many cases presenting with *Nocardia otitidiscaviarum* infections occurred in older adults and immunocompromised individuals receiving chemotherapy or steroids [4-5]. However, a case involving a young female without apparent comorbidities was reported in Turkey [19]. *Nocardia otitidiscaviarum* infection presents similarly to other *Nocardia *species, primarily affecting the lungs, followed by the skin and brain. Lung involvement typically manifests as cavitated masses and consolidations, with pleural involvement potentially leading to empyema. Rare cases of retroperitoneal and vascular involvement have also been reported [19,20]. Diagnosis is made by identifying acid-fast filamentous organisms in sterile samples such as pleural fluid. The gold standard for speciation is 16S RNA analysis or cultures using MALDI-TOF, as in our case.

Optimal antimicrobial treatment for *Nocardia otitidiscaviarum* is not well established due to limited data. Resistance to different antibiotics varies widely, making susceptibility testing essential. Table 3 summarizes the susceptibility of *Nocardia otitidiscaviarum *isolates to common antibiotics based on different studies. Susceptibility to trimethoprim-sulfamethoxazole varies from 50% to 87%. *Nocardia otitidiscaviarum* is mostly susceptible to amikacin and linezolid, whereas resistance to imipenem is high, reaching up to 100%. Similarly, ceftriaxone and ciprofloxacin are generally ineffective against this species.

**Table 3:** Susceptibility of Nocardia otitidiscaviarum to common antibiotics based on different studies TMP-SMX: trimethoprim-sulfamethoxazole; NA: not available

First author	Year	Country	Percentage of *Nocardia otitidiscaviarum* isolates susceptible to antibiotics (%)					
			TMP-SMX	Amikacin	Linezolid	Imipenem	Ceftriaxone	Ciprofloxacin
Hamdi [2]	2020	USA	87	100	100	3	0	0
Harris [7]	2021	USA	100	100	100	0	0	NA
Mctaggart [8]	2015	Canada	83	100	100	0	0	0
Valdezate [9]	2017	Spain	86	97	100	17	0	60
Wei [10]	2017	China	100	100	100	0	0	0
Wang [11]	2022	China	100	100	100	4	4	46
Lu [12]	2020	China	>90	>90	100	40-60	20-40	20-40
Toyocawa [13]	2021	Japan	73	100	100	0	0	0
Gnanam [14]	2020	India	67	100	NA	NA	0	100
Kudru [15]	2021	India	NA	100	100	NA	NA	NA
Rahdar [16]	2021	Iran	50	100	100	0	0	0
Sánchez-Herrera [17]	2012	Mexico	NA	NA	NA	NA	NA	NA
Baio [18]	2013	Brazil	NA	NA	NA	NA	NA	NA

Therefore, amikacin and linezolid should be considered as first-line therapy for treating *Nocardia otitidiscaviarum* infections, pending susceptibility results, with close monitoring of potential renal and myelotoxicity. Trimethoprim-sulfamethoxazole should also be considered a viable option, except in regions with reports of high resistance rates. Linezolid was avoided in our case due to the patient’s pancytopenia and risk of myelosuppression. *Nocardia* infections often require prolonged treatment, generally lasting six to 12 months, depending on the severity and location of the infection.

## Conclusions

*Nocardia otitidiscaviarum* is a rare pathogen that can cause both localized and disseminated disease, similar to other Nocardia species. Its susceptibility to various antibiotics is variable, making susceptibility testing essential when available. Prolonged treatment durations are often necessary, and clinicians should be aware of potential toxicities associated with long-term antimicrobial therapy. Given advancements in diagnostic methods, more cases of Nocardia infections have been identified worldwide over the past decade. However, data from Latin America remains limited. Epidemiological studies with molecular speciation and susceptibility testing of Nocardia species are needed in this region to optimize treatment.
